# COVID-19 and Computer Audition: An Overview on What Speech & Sound Analysis Could Contribute in the SARS-CoV-2 Corona Crisis

**DOI:** 10.3389/fdgth.2021.564906

**Published:** 2021-03-29

**Authors:** Björn W. Schuller, Dagmar M. Schuller, Kun Qian, Juan Liu, Huaiyuan Zheng, Xiao Li

**Affiliations:** ^1^GLAM – Group on Language, Audio & Music, Imperial College London, London, United Kingdom; ^2^EIHW – Chair of Embedded Intelligence for Health Care and Wellbeing, University of Augsburg, Augsburg, Germany; ^3^audEERING GmbH, Gilching, Germany; ^4^Educational Physiology Laboratory, The University of Tokyo, Tokyo, Japan; ^5^Department of Plastic Surgery, The Central Hospital of Wuhan, Tongji Medical College, Huazhong University of Science and Technology, Wuhan, China; ^6^Department of Hand Surgery, Wuhan Union Hospital, Tongji Medical College, Huazhong University of Science and Technology, Wuhan, China; ^7^Department of Neurology, Children's Hospital of Chongqing Medical University, Chongqing Medical University, Chongqing, China

**Keywords:** corona virus, SARS-CoV-2, COVID-19, computer audition, machine listening, computational paralinguistics

## Abstract

At the time of writing this article, the world population is suffering from more than 2 million registered COVID-19 disease epidemic-induced deaths since the outbreak of the corona virus, which is now officially known as SARS-CoV-2. However, tremendous efforts have been made worldwide to counter-steer and control the epidemic by now labelled as pandemic. In this contribution, we provide an overview on the potential for computer audition (CA), i.e., the usage of speech and sound analysis by artificial intelligence to help in this scenario. We first survey which types of related or contextually significant phenomena can be automatically assessed from speech or sound. These include the automatic recognition and monitoring of COVID-19 directly or its symptoms such as breathing, dry, and wet coughing or sneezing sounds, speech under cold, eating behaviour, sleepiness, or pain to name but a few. Then, we consider potential use-cases for exploitation. These include risk assessment and diagnosis based on symptom histograms and their development over time, as well as monitoring of spread, social distancing and its effects, treatment and recovery, and patient well-being. We quickly guide further through challenges that need to be faced for real-life usage and limitations also in comparison with non-audio solutions. We come to the conclusion that CA appears ready for implementation of (pre-)diagnosis and monitoring tools, and more generally provides rich and significant, yet so far untapped potential in the fight against COVID-19 spread.

## 1. Introduction

The World Health Organisation's (WHO) office in China was first made aware of the previously unknown SARS-CoV-2 “Corona” virus on the last day(s) of the last year. On March 11, 2020, the WHO declared the disease triggered by the virus—COVID-19—as pandemic. The spread of the disease induced by the SARS-CoV-2 or “Corona” virus is assumed to underlie an exponential growth. However, whether there are long-term effects after recovery is yet to be fully researched. In the light of this dramatic spread, one is currently internationally witnessing drastic countermeasures that have not been seen in this form over decades in many countries. These include significant public “shut-down” measures to foster “social distancing” in order to slow down and control further spread.

As research globally is making massive efforts to contribute to better understand and fight the phenomenon from a medical and interdisciplinary point of view, also computer science and engineering in terms of “Digital Health” solutions aim at maximum exploitation of available and realisable means. In particular, in combination with artificial intelligence (AI), one can exploit a powerful tool, which so far has largely been tapped for prediction of COVID-19 spread [cf., e.g., (1)], and computer vision (CV) approaches in the corona context such as for automatic screening for COVID-19 on CT images (2, 3). There is, however, broader belief that also other signals including such from sensors on a smartphone could help even in the diagnosis of COVID-19 (4), e.g., the heart rate sensor.

In the following, we aim to provide an overview on what computer audition (CA), i.e., the application of computing for audio processing including “machine listening,” “computational paralinguistics,” and more general speech and sound analysis, but also synthesis, could contribute in this situation. To the best of the authors' knowledge, this resource is so far not used in practise despite offering a plethora of opportunities in this context.

The remainder of this overview is structured as follows: We first summarise phenomena more and less closely related to the case of COVID-19 that have already been targeted by CA and would be readily available. Examples include automatic recognition of speakers suffering from a cold or wearing a mask, breathing, coughing and sneezing sounds, or recognition of audio in spatial proximity. We then shift to the introduction of concrete use-cases how CA could benefit the ongoing global fight against the corona crisis. Subsequently, we introduce challenges and entry barriers from a technical as well as ethical and societal point of view, and discuss limitations before concluding this overview.

## 2. Computer Audition: Related Phenomena

In the following, we set out by show-casing what CA has already successfully targeted as audio use-cases for recognition, and appears related to the task of interest in this contribution—fighting the ongoing COVID-19 spread.

### 2.1. Speech Analysis

Speech analysis by computational means is highly related to the field of computational paralinguistics (5). The field has several related recognition tasks on offer. These are often well-documented in the framework of competitive challenge events such as the Interspeech Computational Paralinguistics Challenge (ComParE). The latter has—perhaps closest related to the COVID-19 case—in its 2017 edition featured the automatic recognition of speech under cold (6), i.e., automatically recognising speakers affected by a cold from the acoustics of their voice. In the challenge of last year, the continuous assessment of breathing patterns from the speech signal appears relevant (7), e.g., as basis to recognise often witnessed symptoms of short-breathiness and breathing difficulties related to COVID-19. The last ComParE challenge further targets the recognition of speech under mask, i.e., the automatic recognition whether a speaker is wearing a facial protective mask, and the recognition of emotion of elderly, which may become interesting in monitoring the aftermath of social isolation of elderly, as was discussed, e.g., in the U.K. for 3 months. On the age scale's opposite end, toddlers' crying sounds seem to be the only indicator to understand if they are suffering from COVID-19 symptoms. In the ComParE challenge series, infant crying was investigated in 2018 (8), and the valence, i.e., positivity of baby sounds in 2019 (9). As symptoms of COVID-19 can also include lack of appetite, it seems further interesting to reference to the EAT challenge (10): In this event, it was demonstrated that one can infer from audio whether speech under eating and eating sounds indicate eating difficulty and “likability” related to whether one enjoys eating. The assessment of sleepiness—a further symptom of COVID-19—was first featured in ComParE in 2011 (11) as binary task, and as continuous sleepiness assessment on the Karolinska sleepiness scale in 2019 (9). Also pain such as headache or bodily pain can accompany COVID-19; speech under pain has also been shown to be automatically accessible (12, 13). When it comes to individual risk assessment and monitoring, speaker traits may be of interest. High mortality risk groups include the elderly, and a (slightly) higher mortality rate was so far seen in male individuals (14). Age and gender were also shown in the context of ComParE, and can be automatically determined reliably given sufficient speech material (15). A history of health issue can further indicate high risk. A number of health-related speaker state information relevant in this context has been shown feasible such as individuals suffering from asthma (16), head-and-neck cancer (17), or smoking habits (18, 19).

Speaker diarization, i.e., determining who is speaking when, and speaker counting (20) can become of interest in the ongoing social distancing. When it comes to counter measures such as quarantine, or risk assessment of individuals, one could also consider the usage of automatic recognition of deceptive speech when people are questioned about their recent contacts or whereabouts, as their personal work and life interests may interfere with the perspective of being sent to quarantine. Deception and sincerity were targeted in ComParE in 2016 (21). Monitoring well-being of individuals during social distancing and quarantine can further find interest in depression and fear recognition. Both were shown feasible to be assessed from speech in the Audio/Visual Emotion Challenge (AVEC) event series (22) including from speech only at reasonable deviation on a continuous scale.

Generally speaking, speech audio also includes textual cues. Broadening up to Spoken Language Processing (SLP), this can also be of help to gather and analyse information from spoken conversations available in individual communications, news, or social media. For textual cues, this has already been considered (23). From a speech analysis perspective, this includes automatic speech recognition (ASR) and natural language processing (NLP).

### 2.2. Sound Analysis

From a sound analysis perspective, one may first consider such interest for COVID-19 use-cases that are produced by the human body. In the context of COVID-19, this includes mostly the automatic recognition of coughs (24–26) including dry vs. wet coughing (27) and dry vs. productive coughing (28) and sneeze (26), swallowing, and throat clearing (25) sounds—all showcased at high recognition rates. As severe COVID-19 symptoms are mostly linked to developing a pneumonia, which is the cause of most deaths of COVID-19 as suggested by post-mortem biopsies (29, 30), it further appears of interest that different breathing patterns, respiratory sounds, and lung sounds of patients with pneumonia can be observed through CA (31), even with mass devices such as smart-phones (32). Of potential relevance could also be the already possible monitoring of different types of snoring sounds (33), including their excitation pattern in the vocal tract and their potential change over time to gain insight on symptoms also during sleep. Further, highest risk of mortality from COVID-19 has been seen for such suffering from cardiovascular disease followed by chronic respiratory disease. In ComParE 2018, heart beats were successfully targeted from audio for three types of heart status, namely, normal, mild, and moderate/severe abnormality. Hearing local proximity from ambient audio further appears possible (34), and could be used to monitor individuals potentially too close to each other in the “social distancing” protective countermeasure scenarios. 3D audio localisation (35) and diarization further allows for locating previously recognised sounds and attributing them to sources. This could further help in the monitoring of public spaces or providing warnings to users as related to individuals potentially being locally too close with directional pointers. Audio source separation and denoising (36) of stethoscope sounds and audio (37) for clinicians and further processing can additionally serve as tool.

## 3. Potential Use-Cases

Let us next elaborate on use-cases we envision as promising for CA in the context of COVID-19. A coarse visual overview on the dependence of CA tasks and these use-cases is provided in Table 1. Check-marks indicate that the already available automatic audio analysis tasks listed in the left column appear of interest in the three major use-case groups listed in the right-most three columns. Note that these are indicative in nature. Further, to provide an impression of the “readiness,” performance indications are given. For a strict comparability of these, they are only provided for tasks that have been featured in the Interspeech Computational Paralinguistics Challenge (ComParE) series.^1^ Shown are the best results after the challenge including by fusion of best participant systems. Likewise, it is assured that test-set labels were unknown to participants and a strict subject independence and challenging conditions including no ability for “cherry picking” alike preselection of test examples are assured. The results overall show that under realistic conditions, the tasks are handled highly above chance level, yet, clearly below “perfect” recognition.

**Table 1:** Interdependence of computer audition (CA) tasks and potential use-cases in the context of the corona crisis.

**CA task**	**COVID-19 relation**	**Performance**	**Risk assessment**	**Diagnosis**	**Monitoring**
**SPEECH ANALYSIS**					
Age & Gender	L	53.6/85.7% UAR (4 age, 3 gender classes)	√		
Breathing	H	0.778 CC_*r*_ (with breathing sensor in [−1,1])		√	√
Cold	H	71.0% UAR (2 classes: yes/no)		√	
COVID-19	H		√	√	√
Crying (infants)	L	78.6% UAR (3 valence classes)		√	
Deception and sincerity	L	72.1%/.654 CC_*p*_ (2 classes: yes/no/in [0,1])			√
Depression	L				√
Emotion (incl. of elderly)	L	63.8% UAR (mean 2 dimensions × 3 levels)			√
Health state	H		√		√
Lung sounds	H			√	√
Mask	H	82.6% UAR (2 classes: yes/no)	√		√
Pain	L		√	√	
Personality	L	70.4% UAR (mean 5D × 2-classes: ±)	√	√	
Sleepiness	L	72.5% UAR (2 classes)		√	√
SLP	L				√
Speaker count	L		√		√
**SOUND ANALYSIS**					
Coughing (dry, wet, productive)	H			√	√
Cardiovascular disease	L	56.2% UAR (3 classes)	√		√
Diarization	L		√		√
Localisation	L		√		√
Proximity	L		√		√
Sneezing	H			√	√
Snoring	L	58.5% UAR (4 classes)		√	√
Source separation	L		√		√
Swallowing	H			√	√
Throat clearing	H			√	√

*The first column indicates the degree of immediate relation to COVID-19 as high (“H”) or low (“L”). The column performance gives an impression of the reliability; for comparability, this is only shown for tasks featured under the same subject-independent challenging conditions in the Interspeech Computational Paralinguistics Challenge (ComParE)—the number of classes or scale and measure of performance are given together with the best result at the end of the event including by fusion of participating systems. UAR, unweighted average recall, i.e., sum of recall divided by number of classes, CC_r_/CC_p_: Pearon's/Spearman's correlation coefficient. SLP, spoken language processing. Health state encompasses a wider range of health factors such as asthma, head and neck cancer, or smoking habits that can be inferred from speech audio*.

### 3.1. Risk Assessment

A first use-case targets the prevention of COVID-19 spread by individual risk assessment. As shown above, speaker traits such as age, gender, or health state can be assessed automatically from the voice to provide an estimate on the individual mortality risk level. In addition, one can monitor if oneself or others around are wearing a mask when speaking, count speakers around oneself, and locate these and their distance to provide a real-time ambient risk assessment and informative warning.

### 3.2. Diagnosis

While the standard for diagnosis of COVID-19 is currently a nasopharyngeal swab, several other possibilities exist including chest CT-based analysis as very reliable resource as outlined above. Here, we consider whether an audio-based diagnosis could be possible. While it seems clear that such an analysis will not be suited to compete with the state-of-the-art in professional testing previously named, its non-invasive and ubiquitously available nature would allow for individual pre-screening “anywhere,” “anytime,” in real-time, and available more or less to “anyone.” To the best of the authors' knowledge, no study has yet systematically investigated audio from COVID-19 patients vs. highly varied control group data including such suffering from influenza or cold and healthy individuals. Unfortunately, coughing and sneezing of COVID-19 patients does not differ significantly to human perception from “normal” patients. This includes lung and breathing sounds. However, (38) assume that abnormal respiratory patterns can be a clue for diagnosis. Overall, by that, it seems unclear if diagnosis from short audio samples of patients could be directly possible, given that most speech or body sounds are likely not to show significant differences for closely related phenomena such as influenza or cold, but a number of encouraging results show that breathing, coughing, and speech sounds could be suited (39). The current Interspeech 2021 ComParE event therefore features COVID-19 recognition from forced cough and speech.

Rather, we believe that a histogram of symptoms over time in combination with their onset appears highly promising. Table 2 visualises this concept in a qualitative manner by coarse ternary quantification of each symptom or “feature” from a machine learning perspective.^2^ Each of the symptoms in the table can—as outlined above—(already) be assessed automatically from an intelligent audio sensor. In a suited personal application such as on a smartphone or smartwatch, smart home device with audio abilities, or via a telephone service, etc., one could collect frequency of symptoms over time and from the resulting histogram differentiate with presumably high success rate between COVID-19, influenza, and cold. By suited means of AI, a probability could be given to users how likely their symptoms speak for COVID-19. Of particular interest thereby is also the “Onset Gradient” feature in Table 2. It alludes to whether the onset of symptoms over time is gradual (i.e., over the span of up to 2 weeks or more) or rather abrupt (i.e., within hours or a few days only), which can be well-observed by AI analysis in a histogram sequence updated over time. Collecting such information from many users, this estimate for histogram-based diagnosis of COVID-19 can be improved in precision over time if users “donate their data.” In addition, clinicians could be given access to the histogram or be pointed to typical audio examples in a targeted manner remotely that have been collected over longer time to speed up the decision whether the users should go for other more reliable forms of testing. This could help to highly efficiently pre-select individuals for screening.

**Table 2:** Qualitative behaviour of symptoms of COVID-19 vs. cold and influenza (flu): Tentative histogram by symptom (“feature”/“variable”) in ternary quantification [from no/low (“+”) to frequent/high (“+ + +”)].

**Symptom**	**COVID-19**	**Influenza**	**Cold**
Breathing: Dypnea (Shortness)	+ + +	++	+
Breathing: Difficulty	+ + +	++	+
Rhinorrhea (Running nose)	+	++	+ + +
Nasal congestion	+	+	+ + +
Coughing	dry ++	dry ++	+
Sneezing	+	+	+ + +
Sore throat	+	++	+ + +
Pain: Body	+	+ + +	++
Pain: Head (Headache)	++	+ + +	+
Fatigue, Tiredness	mild ++	+ + +	+
Appetite loss	+	+ + +	+
Onset gradient	+	+ + +	+

*Shown is also the symptom gradient onset behaviour. Further frequently related variables include behaviour and personality changes, diarrhoea, fever, sore tongue, or watery eyes, which could partially be assessed also by audio—the latter two rather by physiological and visual sensors, respectively. Assembled from diverse references, the table is indicative in nature on purpose, and more fine-grained quantification could apply*.

### 3.3. Monitoring of Spread

Beyond the idea of using smartphone-based surveys and AI methods to monitor the spread of the virus (40), one could use CA for audio analysis via telephone or other spoken conversation. An AI could monitor the spoken conversations and screen for speech under cold or other symptoms as shown in Table 2. Together with GPS coordinates from smart phones or knowledge of the origin of the call from the cell, one could establish real-time spread maps.

### 3.4. Monitoring of Social Distancing and Effects

Social distancing—in already diagnosed cases of COVID-19 or direct contact isolation of individuals—might lead to different negative side effects. People who have less social connexion might suffer from even a weaker immune system, responding less well to pathogens (41). Especially, the high-risk target group of elderly could even encounter suicidal thoughts and develop depression or other clinical conditions in isolation (42). CA might provide indications about social interaction, exemplary speaking time during the day via phone or other devices, as well as measure emotions of the patient throughout the day or detecting symptoms of depression and suicidal risk (43).

In addition, the public obedience and discipline in social distancing could be monitored with the aid of CA. AI allows to count speakers, locate them and their potential symptoms as reflecting in the audio signal (cf. Table 2), and “diarize” the audio sources, i.e., attribute which symptoms came from which (human) individual. Likewise, public spaces could be empowered by AI that detects potentially risky settings, which are overcrowded, under-spaced in terms of distance between individuals, and spot potentially COVID-19 affected subjects among a crowd, and whether these and others are wearing a protective mask while speaking.

### 3.5. Monitoring of Treatment and Recovery

During hospitalisation or other forms of treatment and recovery, CA can monitor the progress, e.g., by updating histograms of symptoms. In addition, the well-being of patients could be monitored similarly to the case of individual monitoring in social distancing situations as described above. This could include listening to their emotions, eating habits, fatigue, or pain, etc.

### 3.6. Generation of Speech and Sound

While we have focused entirely on the analysis of audio up to this point, it remains to state that there may be also use-cases for the generation of audio by AI in a COVID-19 scenario. Speech conversion and synthesis could help those suffering from COVID-19 symptoms to ease their conversation with others. In such a setting, an AI algorithm can fill in the gaps arising from coughing sounds, enhance a voice suffering from pain or fatigue and further more by generative adversarial networks (44). In addition, alarm signals could be rendered which are mnemonic and re-recognisable, but adapt to the ambient sound to be particularly audible.

## 4. Challenges

### 4.1. Time

The fight against COVID-19 has been marked by a race to prevent too rapid spread that could lead to peak infection rates that overburden the national health systems and availability of beds in the intensive care units leading to high mortality rates. Further, at presence, it cannot be clearly stated whether or not COVID-19 will persistently stay as disease. However, recent research and findings (45) as well as model calculations indicate that COVID-19 will continue to heavily spread over the next months in different areas of the world. Enhanced social distancing might delay the spreading. Additionally, at the moment there is no solid research available to prove persistent immunity against the virus after an infection with COVID-19. Therefore, the need to apply measures of enhanced risk assessment, diagnosis, monitoring, and treatment is urgently necessary to support the current medical system as well as to get COVID-19 under control.

### 4.2. Collecting COVID-19 Patient Data

Machine learning essentially needs data to learn. Accordingly, for any kind of CA application targeting speech or sounds from patients suffering from COVID-19 infection, we will need collected and annotated data. At present, such data are hardly publicly available for research purposes, but urgently needed. Hence, a crucial step in the first place will be to collect audio data including highly validated such from diagnosed patients and ideally control subjects under equal conditions and demographic characteristics including control data with a rich representation of further respiratory and other diseases.

### 4.3. Model Sharing

In order to accelerate the adaptation of machine learning models of CA for COVID-19, exchange of data will be crucial. As such data are usually highly private and sensitive in nature, the recent advances in federated machine learning (46) can benefit the exchange of personal model parameters rather than audio to everyone's benefit. Likewise, users of according services can “donate their data” in a safe and private manner.

### 4.4. Real-World Audio Processing

Most of the tasks and use-cases listed above require processing of audio under more or less constrained “in-the-wild” conditions such as audio recording over telephone, VoIP, or audio takes at home, in public spaces, or in hospitals. These are usually marked by noise, reverberation, varying distance to microphone(s), transmissions with potential loss, and further disturbances. In addition, given the pandemic character of the SARS-CoV-2 corona crisis, one will ideally need to be robust against multilingual, multicultural, or local speech and sound variability.

### 4.5. Green Processing

Green processing summarises here the idea of efficiency in computing. This will be a crucial factor for mobile applications, but also for big(ger) data speech analysis (47) such as in the case of telephone audio data analysis. It includes conservative consumption of energy such as on mobile devices, efficient transmission of data such as in the above named federated machine learning in order not to burden network transmission, memory efficiency, model update efficiency, and many further factors.

### 4.6. Trustability of Results

Machine learning and pattern recognition methods as used in CA are usually statistical learning paradigms and hence prone to error. The probability of error needs to be (a) estimated, known as confidence measure estimation, and (b) communicated to users of CA services in the COVID-19 context to assure trustability of these methods. One step further is that results should ideally be explainable. However, eXplainable AI (XAI) itself is at this time a young discipline, but provides an increasing method inventory allowing for interpretation of results (48).

### 4.7. Ethics

Many of the above suggested use-cases come at massive ethical responsibility and burden, which can often only be justified in times of global crisis as the current one. This includes mostly many of the above sketched applications of CA for monitoring. Assuring privacy at all times will be crucial to benefit only the goal of fighting COVID-19 spread without opening doors for massive miss-use. At the same time, balancing between individual interests and the beneficence of groups and societies will need to be carefully considered.

In addition, apart from responsible research, it needs to be assured that the data are representative of all users in all use-cases avoiding potential algorithmic bias. Indeed, the suggested CA algorithms could function better for some parts of the population, because algorithms were trained with data from only one subculture due to different access to resources/technologies. As an example, this could create an asymmetry in the detection of symptoms of subparts of a population (inter- and intra-countries). Deploying the same solution at scale would favour certain social groups and disfavour others (49).

Next, one must assure that common points of reference for comparison across studies are given, the aim of an audio task is well-decided upon, results are interpretable, and communicated to all, including in particular communication of potential limitations (50). Further concerns in this context will discuss legal and societal implications. All of these cannot be discussed here—rather, we can provide pointers for the interested reader as starting points (50–55).

While the technology seems ripe for application, one may ask if we should use it? Or, are the ethical questions that rise from these technologies enough to pause the development of the suggested CA techniques at scale and think on the ethical solutions first? And, do we have enough ethical knowledge today to put enough constraints on the suggested CA applications to make them secure/ethical? These questions touch upon many actions that have been taken during the ongoing pandemic, but of course, this will not justify risking massive personal data leakage or restrictions of personal rights due to missclassifications by AI or more specifically CA. Decisions will need to be made individually per use-case and potential CA solution carefully weighing benefit against risk.

## 5. Limitations

Following the described advantages and the potential uses of these technologies in the case of COVID-19, we now provide a critical thinking about their limitations and discussion about their usability in the described use-cases.

First, as to the tasks described in Table 1, in a non-negligible number of cases the data used for the experiments are still simulated. Hence, the extrapolation from this to real-world scenarios is far from being trivial. Also, in all use-cases, we assumed that there is access to ideal sound recordings so one can track a person at home and in public spaces. Today, cities and homes are not equipped with microphone networks, so smartphones are the preferred choice. On the one hand, these recordings are potentially extremely noisy, reverberated, and marked by package loss, which limits the applicability of the previous research; on the other hand, most use-cases assume that the smartphone is constantly listening, and that AIs are able to detect, for example, if a person is eating (even before wondering if they are eating with appetite or not). Furthermore, it may appear difficult to see how we can envision the application for locating and detecting sound sources from recordings made by smartphones.

Few or none of the technologies mentioned are fully operational today, so we can use them effectively for the proposed objectives (as opposed to computer vision which is already commonly used). And if time is indeed a challenge in this period, it seems that the time necessary to exploit CA efficiently for the COVID-19 task could be the biggest challenge, as software development, deployment, maintenance, testing including medical such, and alike are usually very demanding in time.

Further, it has been noticed that only some specific elements are directly related to COVID-19 (such as pre-diagnosis of COVID-19 from breathing, coughing, or speech). In particular, for the various paralinguistis recognition tasks, these would otherwise further include lung sounds as compared to others diseases such as common cold/influenza. An indication on the degree of immediate relation to COVID-19 is provided in Table 1. On the other hand, many of the introduced aspects bear interest even from monitoring of cold/influenza and other respiratory or even related viral diseases perspective.

Also, in many of the cases described, another simpler means can certainly be used instead to arrive at the same information. For example, bio-signals allow a more direct and more efficient measurement of a person's state of health and its evolution; smartphones and GPS tracking are very effective in locating individuals. Hence, a multi-modal combination of audio and other modalities appears very promising depending on the individual requirements and settings.

In Table 3, we hence investigate whether the suggested CA applications would work better than those that are already implemented at scale or in high technology-readiness state. On a similar line, we indicate where the mentioned CA techniques could complement the current monitoring methods particularly well. We provide the most common alternative methods for the three major use-cases risk-assessment, diagnosis, and monitoring as introduced in section 3. Other alternatives are recently developed and need to be related in a similar fashion to CA (56), once being ready for usage at scale. Also note that the table merely presents a coarse indication. It will depend on the detailed usage which approach is to be preferred. For example, the indicated equality in effort for contact tracing by bluetooth or alike vs. CA is a coarse estimate as, on the one hand, a high cost for development, advertisement, and distribution of such solutions is required. On the other hand, a centralised service based on CA will also come at a high effort: CA population tracking could be extremely expensive in terms of resources and development. Indeed, application development, server infrastructure, data analysis centers, data encryption, storage, and anonymisation as well as all the costs of maintaining these services could easily add up transforming CA solutions in over-priced servers difficult to maintain and without the certainty that they will detect large numbers of COVID-19 cases.

**Table 3:** Nine key aspects: Promising complimentarity of CA with other methods, tentative advantages (“+”) vs. disadvantages (“-”) or equality (“0”) of using audio as the modality as compared to other more established methods used at scale in the medical and related setting.

**Task**	**Method**	**Complementary**	**Reliability**	**Robustness**	**Speed**	**Effort**	**Area covered**	**Accessibility**	**Privacy**	**Cooperation requirement**	**Informationrichness**
Risk assessment	5G/BT contact tracing	√	−	−	+	0	+	+	−	+	+
	Temperature measurement	√	+	+	0	0	+	0	−	−	+
Diagnosis	Blood sample, chest x-ray, heart-rate sensing, upper respiratory sample	√	−	−	+	+	+	+	−	0	+
Monitoring: spread,	Wastewater tracking		0	+	+	0	+	−	−	+	
social distancing,	Video-based	√	−	+	0	+	+	+	0	−	0
	Chip-based		−	−	0	0	+	+	−	+	+
distancing effects,	Analysis by physiology	√	+	+	0	+	+	+	−	+	+
	Analysis by text		0	−	0	−	−	0	−	0	+
	Analysis by video	√	0	0	0	+	+	+	0	−	0
Treatment/recovery	Medical testing		−	−	+	+	+	+	−	0	0
	Human assessment		0	−	0	+	0	+	+	0	−

*Obviously, in general, audio-based assessment requires presence of audio in the first place, which can be positive in terms of privacy, but negative for continuous assessment or such without cooperation requirement. BT, bluetooth*.

As to the alternatives to CA by use-case, for risk assessment, this is currently mainly achieved by the named contact tracing apps run on one's smart phone by bluetooth or 5G methods (57). When active on the phones of two individuals in sufficiently close proximity such as less than 2 m for a minimum set time of more than 15 min, the contacts are stored (usually only locally in anonymous ways). Users that report COVID-19 positive diagnosis are informed back to the service in anonymous forms. Such an approach has been used already for other infectious diseases (e.g., for HIV or tuberculosis). Another increasingly used method is thermal camera based body temperature measurement (58) often in the context of access, which has also been vastly used before, e.g., at airports. A single thermal camera can be used together with subject tracking to assess many individuals by a single device.

For diagnosis, the common present alternatives used at scale are upper respiratory samples such as regarding reverse transcription-polymerase chain reaction (RT-PCR), and blood samples (58), or, chest X-ray. A mobile health alternative is found by intelligent heart rate analysis such as from wrist-worn devices (59).

Monitoring of spread can alternatively, for example, be fulfilled by analysing wastewater (60).

Monitoring of social distancing is realisable also by video-based tracking (61) of individuals or chip-based such as via 5G, bluetooth, or NFC and related technologies, as used, e. g., in factories (57) usually requiring each participant to be equipped, accordingly. Monitoring of social distancing effects is largely related to affective, behavioural, and social computing in more general, for which there exist a range of other modalities—mainly physiology, text, or video (51). For monitoring of social distancing treatment and recovery, mainly the usage of medical testing and mere human assessment form major alternative options.

How CA or CA combinations would compare (e.g., in terms of false positives/false negative rates or detection time) to the already implemented medical and alternatives systems in society will need to be broken down in detail. For instance, questions such as do we have any clues to think that CA will be more robust in terms of diagnostic than the current medical monitoring system will need careful further investigation.

Besides such more technical questions, practical questions on acceptance will also largely dominate the usefulness of CA methods for COVID-19. The tracing application experience has in some countries shown that the population was not fully ready to give away their data. Similar of even bigger societal challenges and limitations of the deployment of CA applications in the context of COVID-19 at scale need to be expected.

## 6. Discussion

In this short overview, we provided pointers toward what CA could potentially contribute to the ongoing fight against the world-wide spread of the SARS-CoV-2 virus known as “corona crisis” and the COVID-19 infection triggered by it. We have summarised a number of potentially useful audio analysis tasks by means of AI that have already been demonstrated feasible. We further elaborated use-cases how these could benefit in this battle, and shown challenges arising from real-life usage. The envisioned use-cases included automated audio-based individual risk assessment, audio-only-based diagnosis directly from speech or (forced) cough-sounds and by symptom frequency and symptom development histograms over time in combination with machine learning, and several contributions to monitoring of COVID-19 and its effects including spread, social distancing, and treatment and recovery besides use-cases for audio generation. At the time of writing, it seems that what matters most is a rapid exploitation of this largely untapped potential. Obviously, in this short overview, not all possibilities could be included, and many further potential use-cases may exist. We also showed key limitations, but others will exist. Further, the authorship is formed by experts on CA, digital health, and clinicians having worked with COVID-19 infected patients over the last months—further insights from other disciplines will be highly valuable to add. The corona crisis demands for common efforts on all ends—we truly hope computer audition can add a significant share to an accelerated success of the crisis' defeat.

## Author Contributions

BS wrote the manuscript. All authors contributed ideas and input and read over the manuscript.

## Conflict of Interest

BS and DS were employed by the company audEERING GmbH. The remaining authors declare that the research was conducted in the absence of any commercial or financial relationships that could be construed as a potential conflict of interest.

## Footnotes

^1^ http://www.compare.openaudio.eu

^2^based on https://www.qld.gov.au, https://www.medicinenet.com/, https://www.medicalnewstoday.com, all assessed on March 20, 2020.
